# Human Miro Proteins Act as NTP Hydrolases through a Novel, Non-Canonical Catalytic Mechanism

**DOI:** 10.3390/ijms19123839

**Published:** 2018-12-02

**Authors:** Daniel T. Peters, Laura Kay, Jeyanthy Eswaran, Jeremy H. Lakey, Meera Soundararajan

**Affiliations:** 1 Institute for Cell and Molecular Biosciences, Newcastle University, Framlington Place, Newcastle upon Tyne NE2 4HH, UK; daniel.peters@newcastle.ac.uk (D.T.P.); jeremy.lakey@ncl.ac.uk (J.H.L.); 2 Department of Applied Sciences Faculty of Health and Life Sciences, Northumbria University, Newcastle upon Tyne NE1 8ST, UK; laura.kay@northumbria.ac.uk; 3 Northern Institute for Cancer Research, Newcastle University, Herschel Building, Newcastle upon Tyne, NE1 7RU, UK; jeyanthy.eswaran@newcastle.ac.uk

**Keywords:** Miro, atypical GTPases, monomeric GTPase, GTPase mechanism, mitochondrial movement, NTPase

## Abstract

Mitochondria are highly dynamic organelles that play a central role in multiple cellular processes, including energy metabolism, calcium homeostasis and apoptosis. Miro proteins (Miros) are “atypical” Ras superfamily GTPases that display unique domain architecture and subcellular localisation regulating mitochondrial transport, autophagy and calcium sensing. Here, we present systematic catalytic domain characterisation and structural analyses of human Miros. Despite lacking key conserved catalytic residues (equivalent to Ras Y32, T35, G60 and Q61), the Miro N-terminal GTPase domains display GTPase activity. Surprisingly, the C-terminal GTPase domains previously assumed to be “relic” domains were also active. Moreover, Miros show substrate promiscuity and function as NTPases. Molecular docking and structural analyses of Miros revealed unusual features in the Switch I and II regions, facilitating promiscuous substrate binding and suggesting the usage of a novel hydrolytic mechanism. The key substitution in position 13 in the Miros leads us to suggest the existence of an “internal arginine finger”, allowing an unusual catalytic mechanism that does not require GAP protein. Together, the data presented here indicate novel catalytic functions of human Miro atypical GTPases through altered catalytic mechanisms.

## 1. Introduction

Mitochondria are highly dynamic organelles that play a central role in multiple cellular processes, including eukaryotic energy metabolism and cell survival [1,2]. The morphology and cellular distribution of mitochondria constantly undergo processes of fusion, fission and cytoskeleton-dependent transport to maintain normal cell function and homeostasis [3,4,5]. The perturbation of these processes leads to a number of neurodegenerative, neuropsychiatric and metabolic disorders [4].

**Figure 1:**
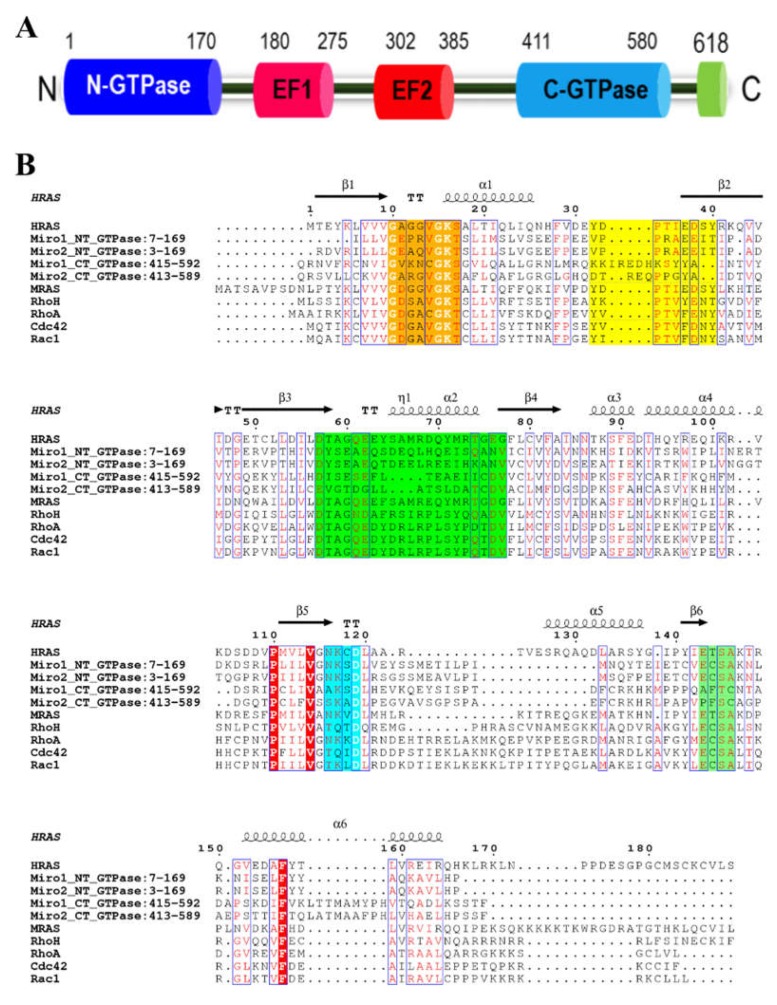
Domain organisation and sequence alignment of Miro proteins. (**A**) Cartoon showing the domain structure of Miro proteins, with the N-terminal GTPase domain, EF hands, C-terminal GTPase domain and C-terminal membrane anchor depicted as cylinders. (**B**) Sequences of GTPase enzymes, as well as the N- and C- terminal GTPase domains of the Miro1 and Miro2 enzymes were aligned using the Clustal Omega webserver and visualised using the ESPRIPT webserver. The G1 loop is highlighted in orange, the G2 loop (Switch 1 region) is highlighted in yellow, the G3 loop (Switch 2 region) is highlighted in green, the G4 loop in cyan and the G5 loop in light green. The secondary structure of H-Ras is depicted above the alignment and conserved amino acids are shown in white, highlighted with a red background if outside of one of the coloured loop regions. Regions of similarity are bounded by blue boxes with similar amino acids shown in red.

Amongst the many cellular GTPases there exist two isoforms of “atypical” Rho GTPases, named mitochondrial Rhos (Miro), have been established to play critical roles in mitochondrial transport, morphology, and homeostasis [6,7]. Mitochondrial Rho GTPases Miro1/RhoT1 and Miro2/RhoT2 are atypical by their primary structure, domain architecture and subcellular localisation in comparison to the Ras superfamily or heterotrimeric GTPases. Miro proteins display a unique domain architecture, with a single polypeptide chain comprising two different GTPase/GTPase like domains and calcium binding EF hands, along with an extreme C-terminal transmembrane domain that acts as an outer membrane anchor (Figure 1A). 

The Miro proteins are conserved across eukaryotic species [8], and mouse model studies suggest human Miro 1 and 2 are functionally distinct [9]. Initial work revealed the indispensable role of the Miro proteins in mitochondrial transport along the microtubule, interacting with the dynein and kinesin motor complexes containing cargo adapter proteins i.e., MILTON in *Drosophila* and TRAK1/2 in humans respectively [10]. When intracellular levels of calcium are high, the EF hand domains of the Miro proteins are saturated, resulting in the dismantling of the kinesin mediated transport motors used for mitochondrial motility, thereby regulating mitochondrial motility [11].

In addition, further studies on the molecular functions of Miro proteins revealed their role in developmental processes, neurological disorders, cell migration and susceptibility to microbial infections [8,12,13]. Recent developmental studies have also revealed that Miro-mediated mitochondrial trafficking is key to the aggregation of germinal granule components during primordial germ cell formation in *Xenopus* embryos [14]. Moreover, Miro1 is a key substrate of an E3 ubiquitin ligase, Parkin, which facilitates proteasomal degradation of Miro in the context of mitochondrial damage and motility [15,16]. A Miro1 C-terminal GTPase (CT-GTPase) domain lysine is necessary and sufficient for efficient ubiquitination by the human E3 ubiquitin ligase Parkin, which is implicated in Parkinson’s Disease [4,17,18]. 

Studies on the N-terminal GTPase domain of *Drosophila* or vertebrate Miros have revealed a conserved role for Miro proteins in the determination of mitochondrial size, distribution and transport across the species [12,19]. Loss of function analysis of the N-terminal GTPase domain showed accumulation of mitochondria in the soma of axons and dendrites of the larval motor and sensory neurons [19,20]. In agreement with these findings, the constitutively active mutant of *Drosophila* Miro (dMiro) (GV13), which contains a mutation in its N-terminal GTPase domain equivalent to the well-established and analogous G12V constitutively active Ras mutant, showed neomorphic phenotypic effects that are reported to be unrelated to GTPase function [12]. In contrast, when the functional effect of the catalytic activity of the CT-GTPase domain was studied using dMiro through a loss of function mutant (T460N), it was clearly shown that the mutation did not impair viability, mitochondrial size, or the distribution of mitochondria. Similarly, the constitutively active CT-GTPase mutant revealed the neotropic effects to be unrelated to the function of the catalytic domain.

The exclusive cellular role of the Miro CT-GTPase domain came to light following the analysis of the formation of the endoplasmic reticulum mitochondria encounter structure (ERMES) complex, which tethers the ER to the mitochondrion [21]. The formation of the complex involves phospholipid exchange between the two organelles. The yeast homologue of Miro, Gem1, is an integral component of ERMES, regulating both the size and number of the complexes. It was established that the CT-GTPase domain was specifically involved in phospholipid exchange, while the N-terminal GTPase domain was involved in the physical association of Gem1 with the ERMES complex [21]. This evidence further demonstrates the non-redundant functions of the Miro protein GTPase domains.

Here, we performed an *in-vitro* characterisation of the catalytic activity of the GTPase domains of the two human Miro proteins. The N-terminal GTPase domain displays GTPase activity despite the absence of key Ras residues including Y32, T35, G60 and Q61. Surprisingly, when the catalytic ability of the previously reported “relic” C-terminal GTPase domains of Miro1 and Miro2 was analysed, they were seen to be active. Moreover, they displayed substrate promiscuity, with hydrolytic activity against ATP and UTP observed in addition to GTP, demonstrating that these domains function as NTPases rather than archetypal GTPases. Furthermore, structural modelling and analysis revealed the Miro proteins have unusual structural features that may function as an ‘internal arginine finger’ that enables unusual transition state and nucleotide hydrolysis. Together, the data presented here shows the novel catalytic functions of this atypical GTPase through altered catalytic mechanisms.

## 2. Results and Discussion

The primary sequences of the GTPase domains of both Miro1 and Miro2 resemble the G domains of classical Ras superfamily proteins (Figure 1B). For the Miro proteins, the G1, G4, and G5 loop sequence motifs are conserved, while the Switch I (G2) and Switch II (G3) regions, which are responsible for Mg^2+^ binding, guanine nucleotide binding and hydrolysis [22,23], show significant differences, most notably in the key catalytic Ras residues (Y32, T35, G60 and Q61). As several mitochondrial functions mediated by Miro proteins had been previously attributed to these GTPase domains [16,19], we sought to characterise the hydrolytic capacity of these domains towards GTP. Constructs were generated of the N-terminal GTPase domain (NT-GTPase), the C-terminal “putative GTPase” domain (CT- GTPase), and a longer construct that comprised both EF hands and C-terminal GTPase domain of human Miro GTPases (EF-CT-GTPase). The resulting proteins were expressed and purified, and their hydrolytic capacity towards GTP determined by phosphate release assays in the presence of MgCl_2_, which acts as a co-factor.

### 2.1. Miro1 and Miro2 N-Terminal GTPase Domains Are Catalytically Active

**Figure 2:**
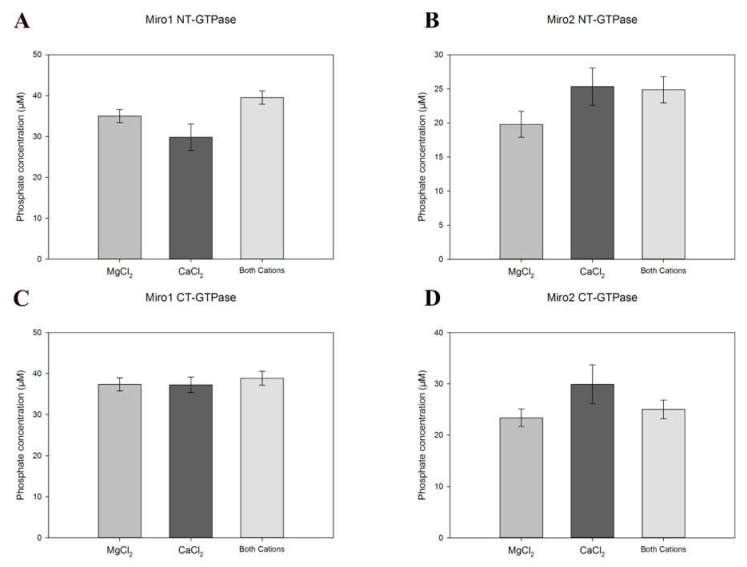
GTP hydrolytic activity of the N- and C-terminal GTPase domains of hMiro1 and 2. Phosphate release as a measure of enzymatic activity determined by the PiColorLock™Gold system (Innova Biosciences, UK) for the N-terminal GTPase domains of Miro1 (**A**) and 2 (**B**), as well as the C-terminal GTPase domains of both proteins (**C**,**D**). Readings are provided as blank-corrected readings. Blank readings comprised assay buffer, cations and GTP. Error bars represent standard error of the mean of 5 experiments.

The NT-GTPases of both Miro proteins showed slow catalytic activity, similar to other monomeric GTPases reported previously [24,25] (Figure 2A,B). As the EF hands present in the Miro proteins can interact with calcium, and the mitochondrial functions of Miro have been shown to be sensitive to calcium levels in the neuronal cells [26], we sought to determine if calcium could substitute magnesium as the co-factor. The assay was therefore repeated in the presence of CaCl_2_, as well as both CaCl_2_ and MgCl_2_. The results showed that there was no selective preference for either of the cations (Figure 2A,B), and that there was no visible additive effect due to the presence of both cations. The catalytic activity was not detected in the presence of EDTA (Figure S1).

### 2.2. C-Terminal GTPase Domains of Miro1 and Miro2 Exhibit Hydrolytic Activity against GTP

The C-terminal putative “GTPase domains” of the human Miros have been previously assumed to be non-catalytic relic domains in the literature [27,28] as the sequence in the highly conserved G2–G5 loops responsible for nucleotide binding and hydrolysis is highly divergent compared with canonical GTPase domains (Figure 1B). Prior to this work, enzymatic assays had not been performed to definitively assess the nucleotide binding or hydrolytic capacity of these domains, but recent studies have shown this “putative GTPase” domain to be a component of the endoplasmic reticulum-mitochondria encounter structure (ERMES) tethering complexes and shown to regulate its numbers and sizes [21]. To assess the GTP hydrolysis capacity of these domains, the assay was repeated with the Miro1 and 2 CT-GTPase proteins in the presence of MgCl_2_, CaCl_2_, or both salts. Surprisingly, the Miro1 and Miro2 CT-GTPase domains were catalytically active and able to hydrolyse GTP, exhibiting a GTPase activity similar to the archetypal Rho GTPases and monomeric Ras Superfamily (Figure 2C,D) [6,29,30]. These results represent the first biochemical evidence of the catalytic functionality of the Miro C-terminal GTPase domains. 

### 2.3. EF Hands do not Influence C-Terminal Domain Catalytic Activity

Miro1 and Miro2 “EF-CT-GTPase” constructs each containing both the EF-hand region and the CT-GTPase domain were then purified (Figure 1A). The crystal structure of the *Drosophila* and human Miro structures clearly show direct interaction between the EF hand and CT-GTPase domain, with the EF hand regions reaching around the transition state stabilising the Switch I region, resulting in a shared interface area of approximately 1500 Å^2^ [31,32]. These constructs were therefore tested for their GTP hydrolytic activity to evaluate whether the EF-hand regions played regulatory roles through intramolecular interactions (i.e., through auto-inhibition or structural obstruction) for the CT-GTPase domain. The results indicated that the CT-GTPase domains of Miro1 and Miro2 are capable of hydrolysing GTP in the presence of MgCl_2_, CaCl_2_ or both cations, regardless of the presence or absence of the EF-hand domains (Figure S2).

### 2.4. The Miro1 and Miro2 C-Terminal GTPase Domains Function as an NTPase, with Propensities towards GTP and ATP as Substrates

Crystal structures of both *Drosophila* Miro and human Miro proteins have revealed that the binding mode of the active site guanine nucleotide of the Miro CT-GTPase domains is similar to that of the Rho and Ras GTPases [31,32]. In both classical human and microbial GTPases, the G4 and G5 regions of the monomeric GTPases have been well established to recognise the guanine nucleotide head group of the GTPase and also regulate GTPase activity through modulating Guanine nucleotide exchange factor (GEF) and effector binding [33,34]. The “classical” binding motifs of the G4 and G5 loops are NKxD and [S/C]A[K/L/T] respectively (where x is any amino acid). However, in the Miro CT-GTPase domains these motifs have mutated to AKSD/SKAD and AFTC/PFSC for the Miro1/Miro2 CT-GTPase domains respectively (Figure 1B). Previous studies have shown mutations in these motifs to have effects on guanine nucleotide recognition and nucleotide turnover [35,36,37], GDP release [38], and the rate of guanine nucleotide exchange [39]. Therefore, we examined the possibility that the Miro CT-GTPase domains may have activities against other NTPs through the use of the PiLock enzyme assay. The PiColorLock™ assay is based on the change in absorbance of the dye malachite green in the presence of phosphomolybdate complexes. PiColorLock Gold unlike other dye-based assays gives a stable endpoint signal and is not prone to precipitation. Moreover, a special stabiliser ensures that the reagent can be used with acid labile substrates.

**Figure 3:**
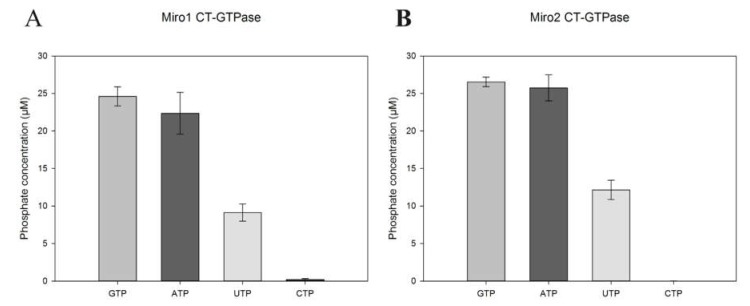
NTP-ase activity of the Miro C-terminal GTPase domains. Phosphate release as a measure of enzyme hydrolytic activity plotted against time determined by the PiColorLock™Gold system (Innova Biosciences, UK) for the C-terminal GTPase domains of hMiro1 (**A**) and hMiro2 (**B**), with GTP, ATP, UTP and CTP used as substrates. Readings are provided as blank-corrected readings. Error bars represent standard error of the mean of 5 experiments.

The results show that the CT-GTPase domains of both Miro proteins displayed catalytic activity towards both ATP and UTP in addition to GTP (Figure 3A,B). This may reflect the atypical nature of the CT-GTPase domains of both Miro proteins. However, no hydrolysis of the pyrimidine nucleotide CTP was observed in any of the conditions tested. It is therefore likely the human Miros CT-GTPase can utilise either GTP, ATP or UTP with similar catalytic efficiency depending on the intracellular nucleotide concentration during rapid mitochondrial movement, fission and fusion.

### 2.5. Rationale for Miro NTP Binding through Structural Modelling

To further understand how these alternate nucleotides bind to active site of the Miro CT-GTPase domains, we performed molecular docking predictions using the SwissDock webserver [40,41] with chain A of the GDP-bound Miro2 CT-GTPase (PDB code: 5KUT) as the target, along with ADP, UDP or CDP as the ligand. The nucleotide di-phosphates were used as ligands in order to simplify the docking process since fewer atoms needed to be modelled, and the experimental structures available for the human Miro proteins were mainly GDP bound. The search area was constrained to the active site by defining a 10 Å^3^ box around the 3′ hydroxyl group of the GDP molecule present in the crystal structure. Models were examined by eye, and the most appropriate models chosen for further analysis based on its similarity to the known bound GDP in terms of the location of the di-phosphate and ribose groups, as well as the number of potential hydrogen bonds seen between the base and the protein. The structure of the Miro2 CT-GTPase is highly similar to that of Miro1 [31] and so it is assumed that the models will be relevant for understanding the nucleotide binding mode of both enzymes. 

**Figure 4:**
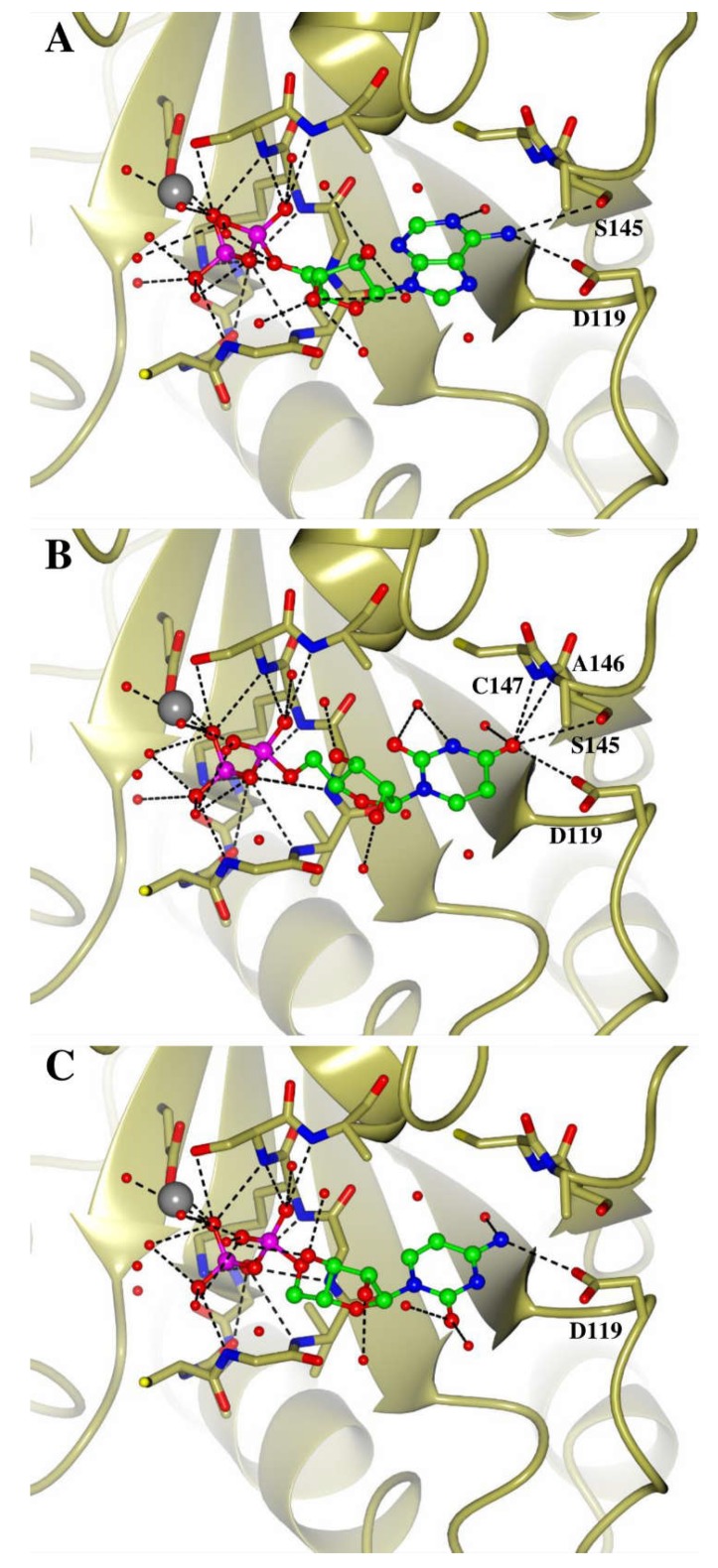
Models of NTP binding by the Miro2 GTPase domain (**A**–**C**) SWISSDOCK models of the Miro2 GTPase domain (PDB: 5KUT) with ADP (**A**), UDP (**B)** and CDP (**C**) docked into the active site. Nucleotides are depicted as balls and sticks, and active site residues shown as cylinders. Atoms are coloured as follows: oxygen is red, nitrogen blue, sulphur yellow, carbon gold, and phosphorous magenta. The active site magnesium is shown as a grey sphere, and solvent molecules are depicted as red spheres. Hydrogen bonds are shown as dashed lines and key residues labelled, with residue numbers corresponding to H-Ras numbering.

The results revealed potential binding modes for all three nucleotides, which utilise the key residues present in the G4 and G5 loops involved in binding the guanine base. For ADP, the phosphate and ribose groups adopt a similar conformation to that seen with GDP, where the adenine base interacts with the protein by forming hydrogen bonds between its amino group and the side chains of S555 and D527 (equivalent to H-Ras residues S145 and D119) (Figure 4A). Likewise, the sugar and phosphate groups of UDP occupy similar positions to those of GDP, and the uracil forms contacts with the backbone amino groups of C556 and A557 (A146 and K147 in H-Ras), as well as the side chain of S555, through its carbonyl group (Figure 4B). In the case of CDP, one potential hydrogen bond could be seen between the amino group and the oxygen of the carboxyl group of D527. This may mean the complex has a lower affinity, suggesting a possible reason for the low level of CTP hydrolysis by the Miro2 CT-GTPase (Figure 4C). The modelling results therefore suggest how the Miro CT-GTPase domains could accommodate these nucleotides, providing a potential explanation for the results of the hydrolysis assays. 

Interestingly, the N-terminal and C-terminal catalytic domains of the human Miro proteins show similar enzymatic hydrolysis in end point assays when tested against GTP. The intracellular concentrations of nucleotides rank in the order of ATP > GTP > UTP > CTP [42]. It is therefore possible that the catalytic domains of GTPases, particularly the entities that function as a part of molecular machinery, might have evolved to utilise any of the higher concentration nucleotides at a given time to serve as efficient motor components regulating mitochondrial motility. This property is essential for the functions of the Miro proteins since at very short notice the mitochondrion needs to be transported through anterograde and retrograde movement to the appropriate position in the neuronal cell or neurone body [MS2] [43,44]. Although the experimental evidence for this broader nucleotide specificity of the tandem domain has been determined in vitro through this study, it is still essential to establish the physiological significance of this in response to cellular cues in vivo.

### 2.6. Structural Comparison with Ras GTPases Suggests that Miro Proteins Utilise a Different Catalytic Mechanism

The two important regions of the Ras GTPases that permit these molecules to function as molecular switches are the Switch I (residues 32–40 in Ras, 34–42 Miro NTD, 448–459 Miro1 CTD, and 446–455 Miro2 CTD) and Switch II (residues 57–76 in Ras, 58–78 in Miro NTD, 476–492 Miro1 CTD and 472–489 Miro2 CTD). While the Switch I region is involved in interactions with downstream effectors and stabilisation of the nucleotide γ-phosphate, the Switch II region participates in nucleotide hydrolysis. The conformational change and stabilisation of these two regions overall dictates the activity of the enzyme, effector binding and nucleotide exchange [45,46]. The canonical model of GTP hydrolysis for Ras-like GTPases involves the conserved residues (numbered according to H-Ras) Y32 and T35 of Switch I, as well as the G60 and Q61 of Switch II. In this model [47], a catalytic water molecule, water 175, is coordinated by residues G60 and T35. It then donates a hydrogen ion to an oxygen of the γ-phosphate group of the GTP nucleotide, with the transition state stabilized through hydrogen bonding interactions with the “bridging” water 189. This water molecule is primed to accept the new hydrogen bond through its pre-existing hydrogen bonding interactions with residues Y32 and Q61. This promotes a partial positive charge, helping to stabilize the negative charge of the leaving group, a function also provided by the GTPase activating proteins (GAPs) [48], which in general fulfil this role through the use of an arginine finger motif [49], which has been implicated to play a crucial role in overcoming the high activation barrier for phosphoryl transfer and bond breaking during hydrolysis. Q61 then moves to interact with the γ-phosphate, releasing an allosteric switch and facilitating the release of the phosphate [47,50]. The importance of these residues results in their high levels of conservation among the Ras type GTPases.

Although both the N- and C-terminal GTPase domains of the Miro proteins display hydrolytic activity towards GTP, examination of their sequences reveals a striking lack of conservation in the aforementioned residues (equivalent to Ras G12, Y32, T35, and Q61), which had led to them being dismissed as relic domains [7] (Figure 1B). To reconcile these two observations, we undertook a structural analysis of the Miro GTPase domains and compared these key loci with their equivalents in the H-Ras GTPase. As no structural data is currently available for the N-terminal GTPase domains, models were generated using the Phyre2 webserver [51]. These models were superimposed onto the GTP-bound form of H-Ras (PDB: 5P21) and the Switch 1 and Switch 2 regions compared.

**Figure 5:**
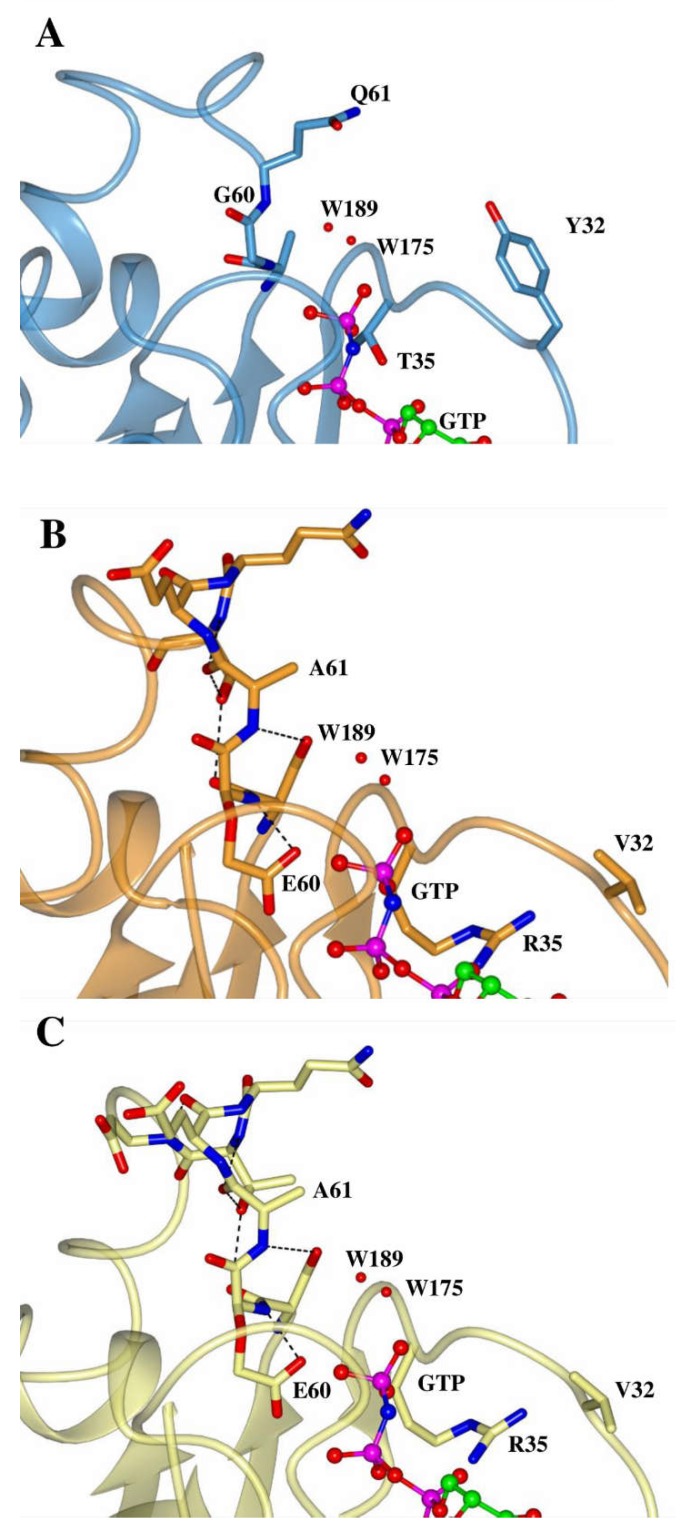
Comparison of the N-terminal GTPase domain models of Miro1 and 2 with H-Ras. Zoomed in views of the Switch 1 and 2 regions of H-Ras (**A**, blue), Miro1 N-terminal GTPase model (**B**, orange) and Miro2 N-terminal GTPase model (**C**, lemon) are shown. GTP and water molecules from the H-Ras structure (PDB: 5P21) are shown as ball and sticks. Key residues are labelled and numbered according to H-Ras, and are shown as cylinders. Atoms are coloured as follows: oxygen is red, nitrogen blue, phosphorous magenta, and carbon according to model colour. Hydrogen bonds are shown as dashed lines, and solvent molecules as red spheres. N-terminal GTPase domain models were made using the PHYRE2 webserver [51].

In the N-terminal GTPase domains of Miro 1 and Miro 2, the residues equivalent to Ras Y32, T35, G60 and Q61 are not conserved and have the following mutations in both the Miro1 and Miro2 NT-GTPase domains: Y32V, T35R, G60E and Q61A (Figure 1B). In our structural model, we see the Miro N-terminal GTPase domains have a valine substituting the otherwise highly conserved tyrosine, resulting in a complete loss of the reactive hydroxyl group (Figure 5). This is of importance since the phosphorylation of this residue on the Switch I effector binding loop can directly modulate effector binding and downstream signalling [52]. Additionally, this substitution would result in the loss of a hydrogen bond to the bridging water 189.

Residue T35 is known to make contacts with the γ-phosphate in the canonical GTPases and its mutation to proline or alanine results in a drastic reduction in nucleotide association capacity [46]. The T35R substitution found in the Miro N-terminal GTPase domains may still preserve hydrogen bonding interactions with water 175. Moreover, it could also potentially form direct contacts with the γ- or β-phosphate (Figure 5).

In the Switch II region, the Miro N-terminal GTPase domains display conservation of the aspartate in the DTAGQ Switch II motif but all the other residues are significantly altered (Figure 1B), resulting in the motif reading DYSEA for both the Miro1 and 2 N-terminal GTPase domains. Here, the conserved threonine is substituted by a much bulkier tyrosine, whereas the alanine is replaced by a more reactive serine residue. The most dramatic effects on the function of these domains likely come from the G60E and Q61A substitutions. The presence of these substitutions in the functional Miro GTPase domains is surprising, as they are critical catalytic residues in the canonical Ras GTPases, and their mutations has been previously linked to oncogenicity [23,53]. The addition of negative charge from the glutamate is likely to induce charge repulsion with the phosphate groups in the position it adopts in the model, and it also clashes with K16 suggesting its orientation could be different to the canonical G60 residue (Figure 5). In the context of the Q61A substitution, it is clear that hydrogen bonds would be lost to both the bridging water 189 and the phosphate leaving group. The expected effect of all these substitutions would therefore be to prevent the canonical GTP hydrolytic mechanism, as the transition state could not be stabilized due to a lack of hydrogen bonding interactions with the bridging water, and the continued maintenance of the allosteric switch. 

**Figure 6:**
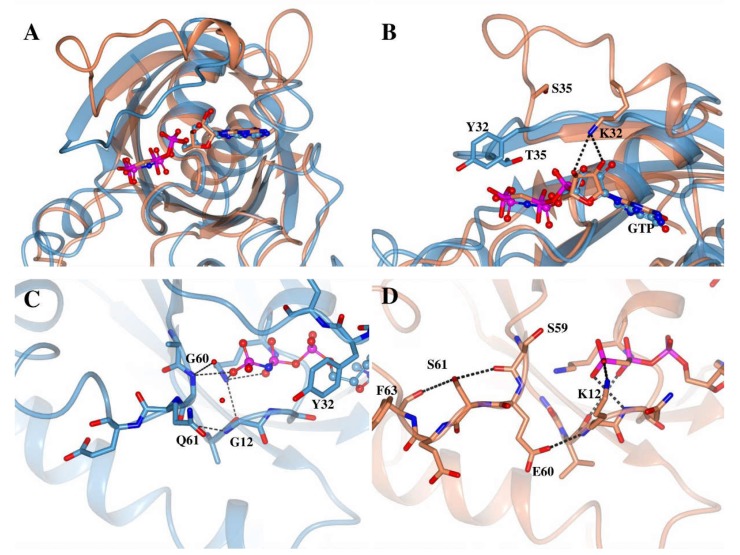
Comparison of the Miro1 C-terminal GTPase domain with H-Ras. Structures of H-Ras (Blue, PDB: 5P21) and the C-terminal GTPase domain of Miro1 (Coral, PDB: 5KSZ) are shown superimposed (**A**) with a close up view on the Switch 1 (**B**), and Switch 2 regions (**C**,**D**). GTP is shown as cylinders for H-Ras, and balls and sticks for Miro1. Key amino acid residues are labelled, numbered according to H-Ras, and are shown as cylinders. Atoms are coloured as follows: oxygen is red, nitrogen blue, phosphorous magenta, and carbon according to model colour. Hydrogen bonds are shown as dashed lines and solvent molecules as red spheres.

Similar to the N-terminal domain, the C-terminal domains of Miro1 and 2 display a completely different pattern of residues to those conserved in the Ras-like GTPase enzymes. Here, Y32, T35, G60 and Q61 are present as K, S, E, S and D, P, D, T in Miro1 and 2 respectively (Figure 1B). To assess the structural effect of these amino acid substitutions, the structure of the C-terminal GTPase domain of Miro1 (PDB:5KSZ) was compared with H-Ras (PDB: 5P21), both in the GTP bound form (Figure 6). Firstly, the entire positioning of the Switch 1 region in Miro1 is remarkable, with a conformation that places it away from the ligand (Figure 6A). Therefore, the equivalent residues in Miro1 already occupy vastly different positions in the structure. The K residue equivalent in the sequence alignment to Y32 appears to fulfil an interesting role, interacting directly with the 2′ and 3′ hydroxyl groups of the ribose (Figure 6B). The T35S change however is of less interest, as it positions the serine away from the nucleotide, with the side chain pointing out into space. The G60E and Q61S substitutions are dramatically different from the H-Ras structure, making no observable contacts with any water molecules, and instead forming an H-bonding network, which may act to maintain the position of the G3 loop of the switch region (Figure 6C,D). Here, E60 forms a hydrogen bond with the backbone of K12, while S61 interacts with S59 and F63 through its side chain. Although the effect of the D32/P35 mutations in Miro2 are difficult to predict without an equivalent GTP bound form structure to compare, the D60/T61 residues are likely fulfil a similar role to those found in Miro1. Therefore, as above, these changes seem to rule out a canonical mechanism being employed in the Miro1 and 2 C-terminal GTPase domains. 

In addition to these observations, another point of interest in the Miro1 C-terminal GTPase structure is the lysine present at position 12 (Figure 6D). Mutations in this position have been shown to be the cause of oncogenic mutations in the Ras family GTPases [54], as steric hindrance prevents hydrolysis. Not only would a bulky lysine residue be expected to produce this same effect, it also appears to interact directly with the γ-phosphate, perhaps giving it a role in stabilizing the leaving group following hydrolysis. Moreover, a difference in the conformation of this residue can be observed when comparing the Miro1 C-terminal GTPase GTP and GDP bound structures. In the GDP bound structure where the γ-phosphate is no longer present, the lysine points away from the active site into space. The residue is present as an alanine in the Miro2 N-terminal GTPase domain, and as a proline in the Miro1 N-terminal GTPase domain and Miro2 C-terminal GTPase domains. Therefore, if this lysine does have a functional role, it is confined to the Miro1 C-terminal GTPase domain only. 

Comparison with other atypical GTPases, Centaurin γ1, RND1 and RND3, reveals that within the common fold, the largest structural difference can be seen in loop G2/Switch I (Figure S3). Here, the loop in Miro2 adopts a conformation that is not seen in the other atypical GTPase structures. On the sequence level, loops G1, G4 and G5 show at least partial conservation of key nucleotide binding residues, consistent with their function, but are variable in loops G2 and G3 (Figure S4). In terms of the critical catalytic residues T35 and G60, the threonine is found in the RND1 and RND3 proteins, but not Centaurin γ1 or the Miro proteins, and the glycine (G60) in Switch II is conserved in all proteins apart from the Miro proteins. These findings suggest that the Miro proteins are unique even amongst atypical GTPases, harbouring substitutions of highly conserved catalytic residues and adopting an unusual G2 loop conformation, whilst still maintaining their hydrolytic capacity. 

### 2.7. The Miro Proteins’ Catalytic Mechanism May Involve an “Internal Arginine Finger”

**Figure 7:**
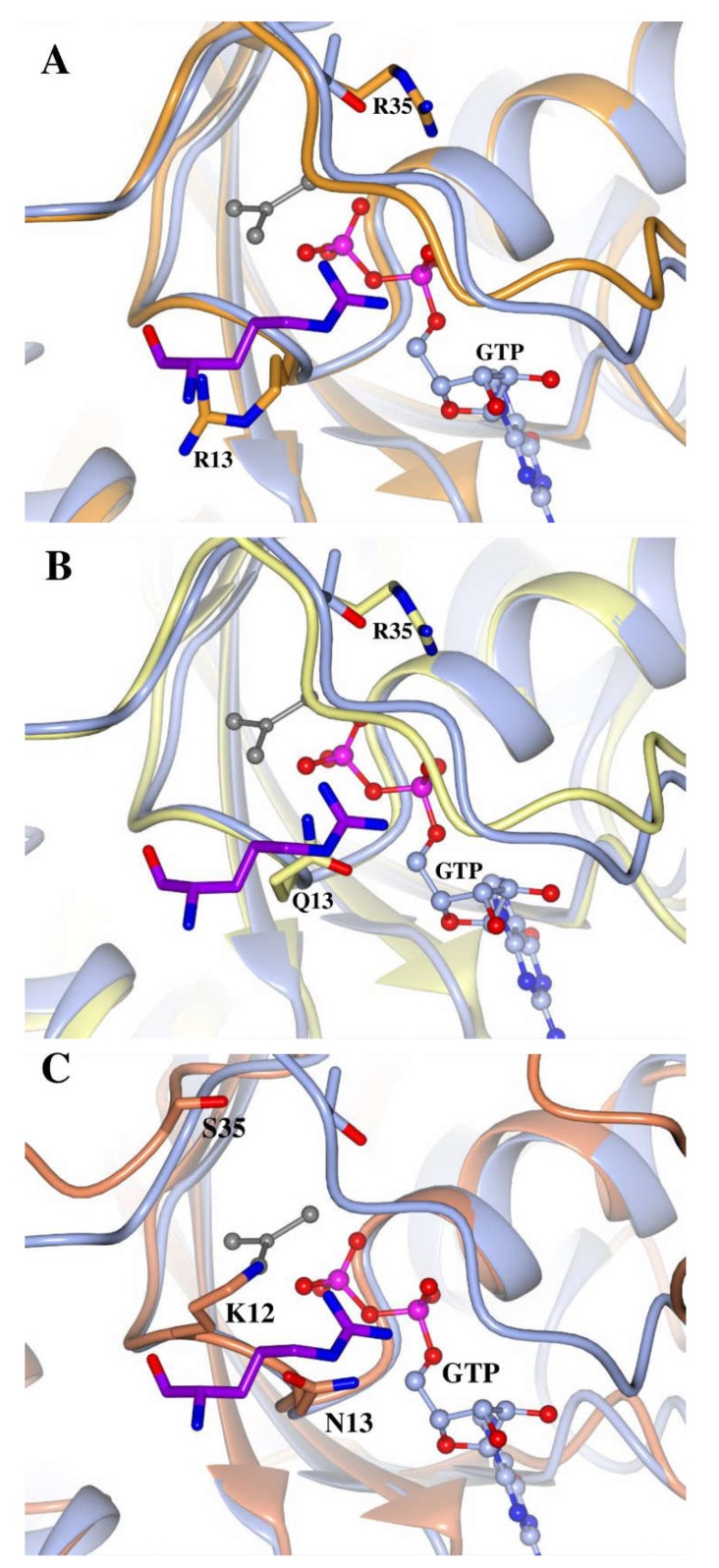
The “internal arginine finger” of Miro1 and 2 GTPase domains. Structures of the H-Ras:RasGAP complex (H-Ras—Blue, RasGAP—purple, PDB: 1WQ1), the models of the N-terminal GTPase domains of Miro1 (orange, **A**), Miro2 (lemon, **B**) and the C-terminal domain of Miro1 (coral, **C**, PDB: 5KSZ) are shown superimposed. The GTP analogue (GDP/AF3) from the H-Ras:RasGAP structure is shown as ball and sticks. Key residues are labelled, numbered according to H-Ras and shown as cylinders. Atoms are coloured as follows: oxygen is red, nitrogen blue, phosphorous magenta, aluminium fluoride is grey, and carbon is coloured according to model. Carbon atoms of the arginine finger of the RasGAP from the H-Ras:RasGAP are highlighted in purple.

The biochemical assays have established the hydrolytic activity of the Miro GTPase domains, but the sequence and structural analysis show clearly that the Miro GTPases cannot utilize the canonical hydrolysis mechanism found in other GTPases, or, indeed, the mechanisms of the atypical GTPase enzymes. Therefore, the mechanism through which hydrolysis occurs remains to be characterised. In the Miro1 N-terminal GTPase model, two arginine residues, one in position 35, the other at position 13, flank the terminal phosphate groups of the nucleotide, with R13 appearing to occupy a similar position to the arginine finger of the RasGAP p120 (PDB: 1WQ1, Figure 7A). Similarly, in the Miro2 C-terminal GTPase domain model, an arginine can be found at position 35, with position 13 occupied by a glutamine residue (Figure 7B); and in the Miro1 C-terminal GTPase domain structure, there is a lysine at position 12, and an asparagine at position 13 (Figure 7C). A previous report has shown the existence of an “asparagine thumb” in the GAP of the Ras homologue Rap1 (which itself lacks a catalytic glutamine residue), where a catalytic asparagine replaces the arginine finger [55]. It may be possible that these residues, not present in the canonical GTPases, could function as an “internal arginine finger”, stabilizing the transition state during nucleotide hydrolysis and allowing faster rates of catalysis without the need for a GAP protein. However, it should be noted that the residues of the C-terminal Miro2 GTPase are not similar in these positions and are unlikely to take this role. Moreover, the plasticity and flexibility of these novel GTPase domains may offer unusual catalytic mechanisms that have yet to be determined. 

From these studies, the Miro proteins have been shown to be a unique subfamily of guanine nucleotide binding protein superfamily that employ substrate promiscuity and novel hydrolytic mechanisms, possibly in order to achieve the required mitochondrial transport. The mitochondrion has evolved to perform diverse functions that are central to a variety of metabolic pathways and signalling processes, including those that control ATP supplies, ion homeostasis and cell death. Therefore, efficient mitochondrial transport is of critical importance to the cell. The unusual catalytic mechanisms the GTPase domains that the Miro proteins appear to employ, along with the observed substrate promiscuities of the CT-GTPase domains, may have evolved to provide an effective molecular basis for this transport.

## 3. Materials and Methods

### 3.1. Cloning of Miro1 and Miro2 Mutants

pRK5-Myc mammalian expression vectors encoding full-length wild type versions of hMiro1 and 2 were kindly donated by the Pontus Aspenström Group at the Karolinska Institutet, Sweden [6]. These vectors were used to sub-clone hMiro1 and hMiro2 (N-terminal GTPase; C-terminal GTPase and EF-CT-GTPase constructs) separately into the bacterial expression vector pET-28a, which contains an N-terminal hexahistidine tag between NdeI/XhoI restriction sites.

PCR fragments were integrated into the vector through ligation using T4 DNA Ligase (NEB) before transformation into chemically competent TOP10 *E. coli*, that were selectively grown on 50 µg/mL kanamycin containing LB-Agar plates. The primers used for cloning in to the bacterial expression hosts are given in Table 1. Plasmid DNA from one clone per construct was confirmed by sequencing using the T7 promoter and T7 terminator sequencing primers, yielding the expected sequence.

### 3.2. Expression and Purification of Miro Constructs

*E. coli* C41 cells freshly transformed with either the Miro N-terminal/C-terminal GTPase domains or the EF-CT-GTPase construct were used for starter cultures that was then used to inoculate large 2-6 litre cultures. Miro protein overexpression was induced using 1 mM IPTG at 18 °C for the N- and C-terminal GTPase constructs and 0.1 mM IPTG for the longer EF-CT-GTPase constructs overnight. Cells were lysed and cell-free supernatant obtained by centrifugation of the solution at 18000× *g*, 4 °C for 1 h. The soluble fraction was then passed through a Ni-NTA column attached to an ÄKTA Prime fast protein liquid chromatography (FPLC) system. The separation was achieved by subjecting the sample to a gradient of imidazole from 50–500 mM in a buffer containing 50 mM HEPES, 500 mM NaCl, 5% glycerol. Fractions containing the proteins of interest were identified by SDS-PAGE analysis, and were pooled and concentrated using Amicon 10 kDa cutoff filters. Proteins were then loaded on to a 16/60 S200 gel filtration column equilibrated in a running buffer containing 25 mM HEPES, 300 mM NaCl, 2% glycerol, 5 mM DTT. The purified recombinant human Miro proteins were then used for further characterisation.

### 3.3. PiColourLock™ Assessment of Phosphate Generation 

PiColourLock™ Gold, a modified form of the standard malachite green assay kit (Innova Biosciences, Cambridge, UK) was utilised as a means of verifying the hydrolytic capacity of target enzymes. A standard curve for phosphate concentration with this kit was generated based on the manufacturer’s instructions. This standard curve was used to assess phosphate generation by target enzymes when assays were performed according to the standard operating procedure supplied by the manufacturer. A typical reaction mixture comprised the following: the enzyme of choice, GTP and one of more cations (2 mM each of MgCl_2_, CaCl_2_ or both cations) and assay buffer (25 mM HEPES pH7.4, 300 mM NaCl, 2% glycerol and 5 mM DTT). After 30 minutes, the reaction was stopped by addition of the “PiColourLock™ Gold Mix”. This plate was shaken gently and incubated for 5 minutes at room temperature prior to addition of the PiColourLock™ stabiliser compound. After 15 minutes, the absorbance of each well was read at 635 nm on a FLUOStar Omega plate reader. Absorbance values were generated by subtracting blank absorbance obtained from test reaction mix containing all components but Miro enzyme from each raw value. Every experiment variant was repeated 3–5 times to determine average release of phosphate from the nucleotides at 25 degrees., and mean values used to generate bar charts, with standard error of the mean calculated for each experiment. 

### 3.4. Structural Modelling and Analysis

Miro1 and 2 N-terminal domain models were produced through input of the amino acid sequence of residues 7–169 of Miro1 and residues 3–169 of Miro2 into the Phyre2 webserver [51], followed by structural analysis using the CCP4MG software package [56]. Both models used the structure of the GTP-bound form of RhoA from a fusion protein (PDB: 5C2K) as a template. NTP docking models were generated using the SwissDock webserver [40,41] with the Miro2 CT-GTPase:GDP complex (5KUT chain A) as the target and either ADP, UDP or CDP as the ligand. To ensure models would be clustered around the active site, the search area was constrained to a 10 Å^3^ box centred on the 3′ hydroxyl group of the bound GDP. Models were initially analysed in UCSD Chimera [57] were compared with the GDP bound Miro2 CT-GTPase structure (5KUT, chain A). Models that had similar phosphate and ribose positions to GDP and had predicted hydrogen bonds with the G4 and G5 loop residues of the protein were swapped with GDP in the .pdb file and opened in the CCP4MG software package [56] for generating figures. Sequence alignments were produced using the Clustal Omega [58] and ESPript 3.0 [59] webservers. 

## Figures and Tables

**Table 1:** Primers used to create Miro1 and Miro2 constructs. Numbers in brackets denote residue numbers.

Clone	Forward Primers	Reverse Primers
Miro1 NT-GTPase (K3-P169)	5′-TGCCATAGCATATGAAGAAAGACGTGCGGAT-3′	5′-TGCCATAGCTCGAGTCAAGGATGAAGAACAGCTTTCTGTG-3′
Miro1 CT-GTPase (Q415-P580)	5′-TGCCATAGCATATGCAAAGAAATGTGTTCAGATGTAATG-3′	5′-TGCCATAGCTCGAGTCACGGATACATGGCCATTGTT-3′
Miro1 EF-CT-GTPase (E177-R590)	5′-TGCCATAGCATATGGAGGAGAAGGAGATGAAACCA-3′	5′-TGCCATAGCTCGAGTCAAGCTCTTGGGGTCAGCTTGT-3′
Miro1 NT-GTPase (R3-P169)	5′-TGCCATAGCATATGCGGGACGTGCGCATCCTGTTA-3′	5′-TGCCATAGCTCGAGTCATGGGATGCAGGACGGCCTT-3′
Miro1 CT-GTPase (Q413-F589)	5′-TGCCATAGCATATGCAGCGGAGCGTCCTCCTGT-3′	5′-TGCCATAGCTCGAGTCAGAAGGAAGAGGGATGCAGCTCT-3′
Miro2 EF-CT-GTPase (E177-P586)	5′-TGCCATAGCATATGGAGGCCAAGCAGTTGAGG-3′	5′-TGCCATAGCTCGAGTCAGGGATGCAGCTCTGCGTG-3′

## Supplementary Materials

Supplementary materials can be found at http://www.mdpi.com/1422-0067/19/12/3839/s1.
