# Supplementary material for: Human Miro Proteins Act as NTP Hydrolases through a Novel, Non-Canonical Catalytic Mechanism
Source: Int J Mol Sci. 2018 Dec 2;19(12):3839. doi: 10.3390/ijms19123839 (PMC6321465; doi:10.3390/ijms19123839)
Supplement: Supplementary file 1 [file ijms-19-03839-s001.pdf]

Supplementary Material

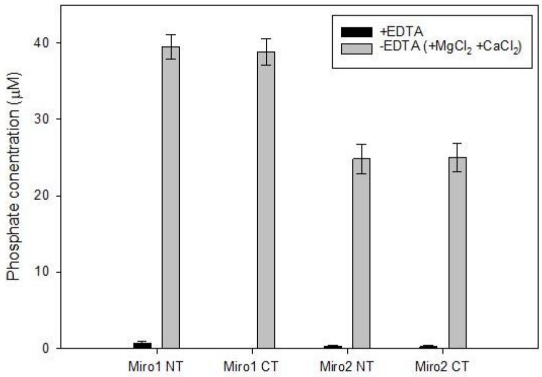

Figure S1. GTP hydrolytic activity of Miro proteins in the presence of EDTA. Phosphate release as a measure of enzymatic activity determined by the PiColorLock™Gold system (Innova Biosciences, UK) for the N-terminal and C-terminal GTPase domains of Miro1 and 2 both in the presence and absence of 2 mM EDTA. Readings are provided as blank-corrected readings. Blank readings comprised assay buffer, cations and GTP. Error bars represent standard error of the mean of 3 experiments.

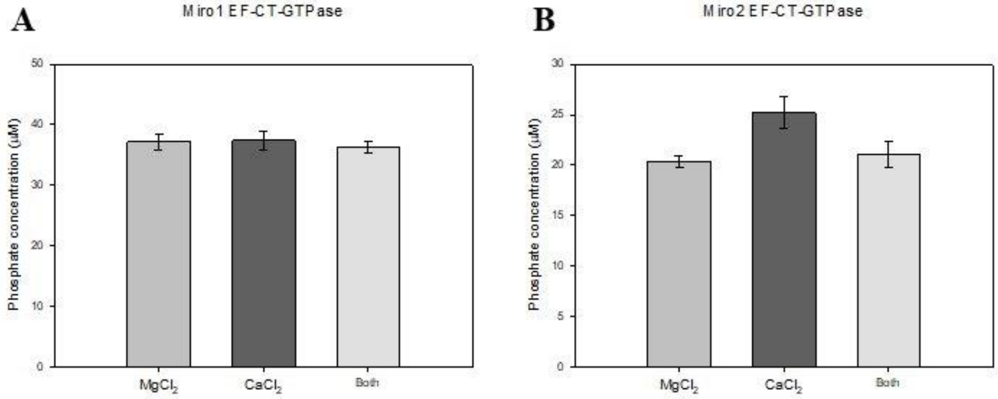

Figure S2. GTP hydrolytic activity of the EF Ct Miro1 and Miro2 proteins.

Amount of phosphate generated determined using the PiColorLock™Gold kit (Innova Biosciences, UK) for the EF-CT-GTPase Miro1 (A) and Miro2 (B) proteins. Readings are provided as blank-corrected readings. Error bars represent standard error of the mean of 5 experiments.

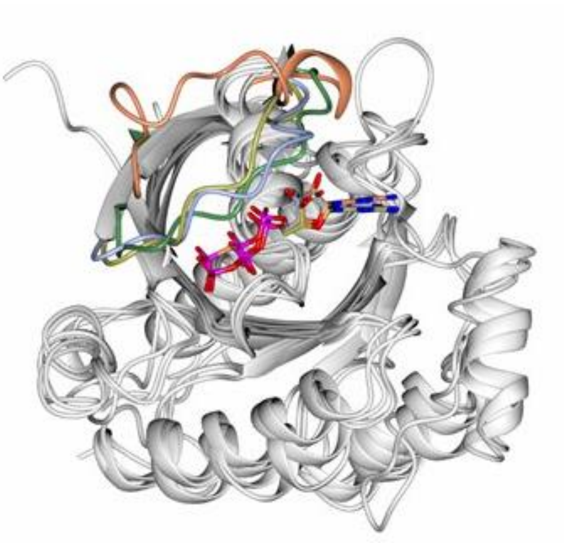

Figure S3. Comparison of the Miro1 C-terminal GTPase domain with atypical GTPases. The Miro1 C-terminal GTPase structure in the GTP bound state (PDB code: 5KSZ) was superposed with the structures of GTP-bound RND1, RND3 and apo-Centaurin γ1 (PDB: 2CLS, 1M7B and 2BMJ respectively). Proteins are depicted in ribbon format, with the G2 loop highlighted in coral for Miro1, blue for RND1, gold for RND3 and green for Centaurin γ1. Bound GTP molecules are shown as sticks, coloured by element (red – oxygen, blue –nitrogen, purple – phosphorous) with carbon atoms shown in the same colour as the corresponding protein G2 loop.

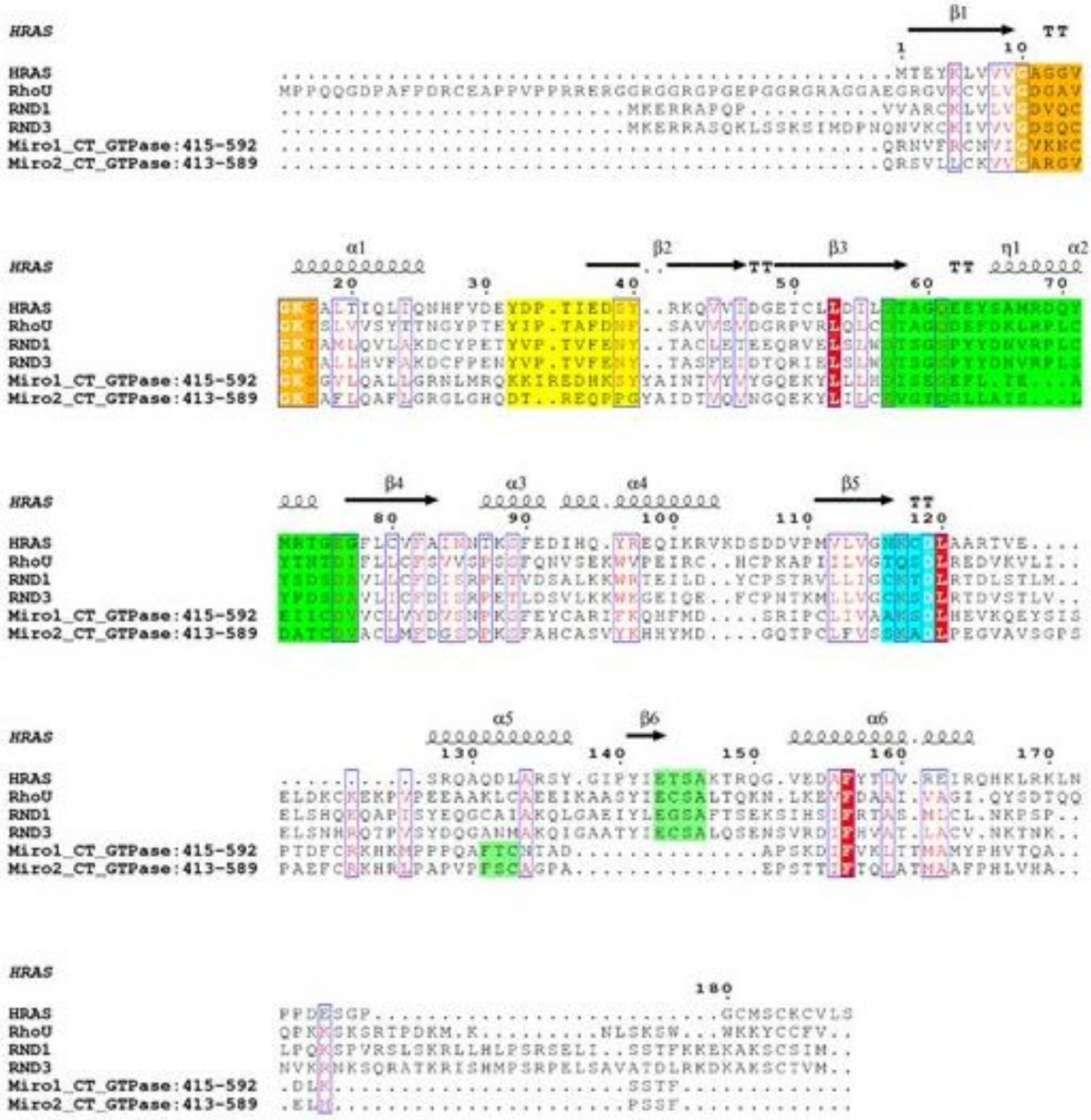

Figure S4. Sequence alignment of atypical GTPase enzymes. Sequences of atypical GTPase enzymes, as well as H-Ras and the N- and C- terminal GTPase domains of the Miro1 and Miro2 enzymes were aligned using the Clustal Omega webserver and visualised using the ESPRIPT webserver. The G1 loop is highlighted in orange, the G2 loop (Switch 1 region) is highlighted in yellow, the G3 loop (Switch 2 region) is highlighted in green, the G4 loop in cyan and the G5 loop in light green. The secondary structure of H-Ras is depicted above the alignment and conserved amino acids are highlighted.
